# Postoperative discovery of molar pregnancy

**DOI:** 10.11604/pamj.2024.47.192.43337

**Published:** 2024-04-16

**Authors:** Haithem Aloui, Hatem Frikha

**Affiliations:** 1 Department ‘C’ of Gynecology and Obstetrics, Tunis Maternity and Neonatology Center, Faculty of Medicine of Tunis, University of Tunis El Manar, Tunis, Tunisia

**Keywords:** Molar pregnancy, uterus, hysterectomy

## Image in medicine

This concerns a 52-year-old patient presenting with abdominal-pelvic pain and diffuse bloating. Upon gynecological examination, an enlarged uterus is noted. Endovaginal ultrasound reveals a 7 cm intracavitary fibroid with suspected degeneration. The patient underwent a hysterectomy. Intraoperatively, the surgeon observes a soft consistency of the uterus and decides to proceed with bilateral annexectomy. Upon dissection of the operative specimen, the typical appearance of a molar pregnancy is noted. This finding is confirmed by anatomopathological examination. The patient had a good postoperative recovery.

**Figure 1:**
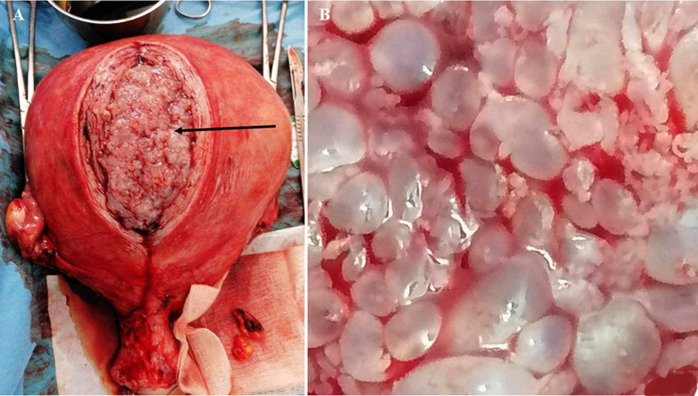
molar pregnancy: A) perioperative cluster of grapes appearance (black arrow); B) macroscopic view

